# Update on left atrial appendage closure for neurologists

**DOI:** 10.1093/esj/aakaf018

**Published:** 2026-01-01

**Authors:** Karl Georg Haeusler, Luciano A Sposato, Marek Grygier, Tatjana Potpara, Jens Erik Nielsen-Kudsk, Lucas V A Boersma, Gregory Y H Lip, Renate B Schnabel, Pavel Osmancik, Boris Schmidt, Wolfram Döhner, Jan Kovac, A John Camm

**Affiliations:** Department of Neurology, Universitätsklinikum Ulm, Ulm, Germany; Department of Clinical Neurological Sciences, Western University, London, Ontario, Canada; Chair and 1st Department of Cardiology, Poznan University of Medical Sciences, Poznan, Poland; Medical Faculty, University of Belgrade, Belgrade, Serbia; University Clinical Centre of Serbia, Belgrade, Serbia; Aarhus University Hospital, Aarhus, Denmark; Cardiology Department, St. Antonius Hospital Nieuwegein/Amsterdam University Medical Centers, Nieuwegein, The Netherlands; Liverpool Centre for Cardiovascular Science at University of Liverpool, Liverpool John Moores University and Liverpool Heart & Chest Hospital, Liverpool, United Kingdom; Danish Center for Health Services Research, Department of Clinical Medicine, Aalborg University, Aalborg, Denmark; Department of Cardiology, University Heart and Vascular Centre Hamburg, Hamburg, Germany; German Centre for Cardiovascular Research (DZHK), Partner Site Hamburg/Kiel/Lübeck, Hamburg, Germany; Department of Arrhythmology, University Hospital Kralovske Vinohrady, Prague, Czech Republic; Cardioangiologisches Centrum Bethanien, Agaplesion Markus Krankenhaus, Frankfurt,Germany; Berlin Institute of Health-Center for Regenerative Therapies, Berlin, Germany; Deutsches Herzzentrum der Charité, Campus Virchow Klinikum, Berlin, Germany; German Centre for Cardiovascular Research (DZHK) - partner site Berlin, Charité - Universitätsmedizin Berlin, Berlin, Germany; Leicester NIHR BRU, University of Leicester, Glenfield Hospital, Leicester, United Kingdom; City St. George’s University of London, London, United Kingdom

**Keywords:** atrial fibrillation, bleeding, left atrial appendage closure, stroke prevention

## Abstract

A significant proportion of patients with atrial fibrillation (AF) who need thromboembolic protection are not treated with or discontinue oral anticoagulation after its initiation. Undertreatment in clinical practice has not improved sufficiently despite the availability of direct oral anticoagulants, which are associated with less intracranial bleeding than vitamin K antagonists. Multiple reasons account for this phenomenon, including bleeding events or ischemic strokes while on anticoagulation, poor treatment adherence despite best educational attempts, or aversion to drug therapy. Percutaneous left atrial appendage (LAA) closure was introduced as an alternative to pharmacological therapy in AF patients in the early 2000s. Due to significant improvements in procedural safety over the years, left atrial appendage closure (LAAC), predominantly achieved through a percutaneous catheter-based device implantation approach, is increasingly favoured for preventing thromboembolic events in patients who cannot achieve effective anticoagulation or have a high hemorrhagic risk. This focused summary and update of a recently published practical guide, developed within guideline/guidance boundaries, provides a perspective of current evidence of potential indications, benefits, complications and limitations of LAAC for neurologists and stroke physicians who may consider this increasingly utilised therapy.

## Graphical Abstract

**Graphical abstract:**
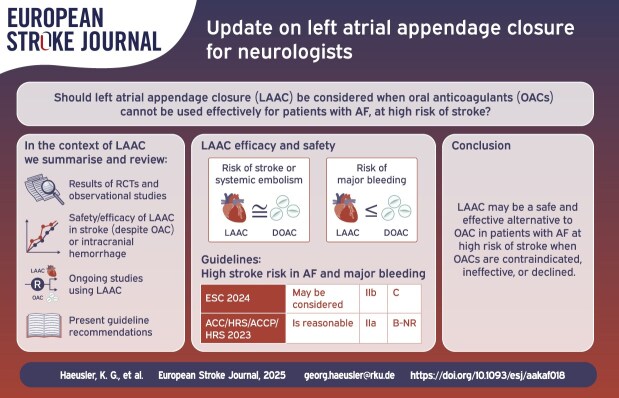

## Introduction

Atrial fibrillation (AF) is the most prevalent sustained cardiac arrhythmia amongst adults, linked to higher morbidity and mortality rates, primarily due to ischemic stroke and heart failure.^1^ The absolute number of people living with AF is progressively increasing due to the overall ageing of the World population.^2^ Stroke reduction in patients with AF requires an integrated care approach, promoted as the evidence-based **Atrial Fibrillation Better Care** pathway in many guidelines globally^3^ and more recently, as AF-CARE in the 2024 *European Society of Cardiology* Guideline,^4^ where the “[A]” refers to “Avoid stroke and thromboembolism”^5^ and SOS in the 2023 ACC/AHA/ACCP/HRS guidelines, where the first [S] is for Stroke—Assess and Treat.^6^ Oral anticoagulants (OACs) such as vitamin K antagonists (VKAs) and direct oral anticoagulants (DOACs) substantially reduce the risk of AF-related stroke. However, they also increase the risk of major bleeding, including ICH (1–3 per 100 patient-years).^7^ In well-managed patients on anticoagulants, the residual risk of stroke is 0.8/100 patient-years.^8^ At present, there is no evidence that factor XI inhibitors, which cause less bleeding, are as effective or better than other anticoagulants for stroke reduction in patients with AF,^9^^,^^10^ although other studies are underway and well advanced.^11^

**Figure 1:**
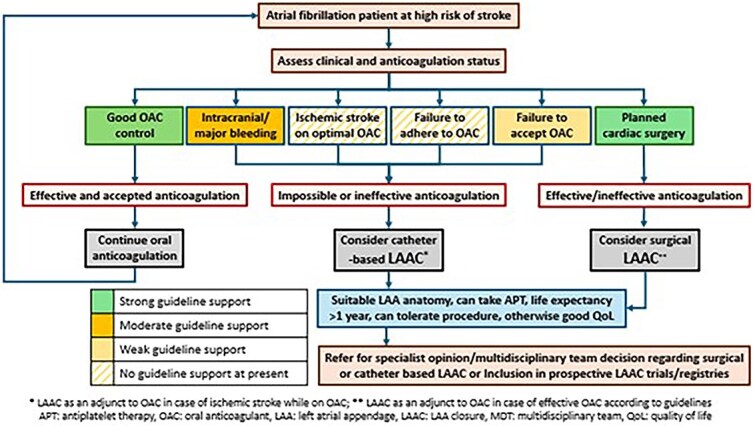
Management strategy for stroke in patients with atrial fibrillation after or at high risk of ischemic stroke.

**Figure 2:**
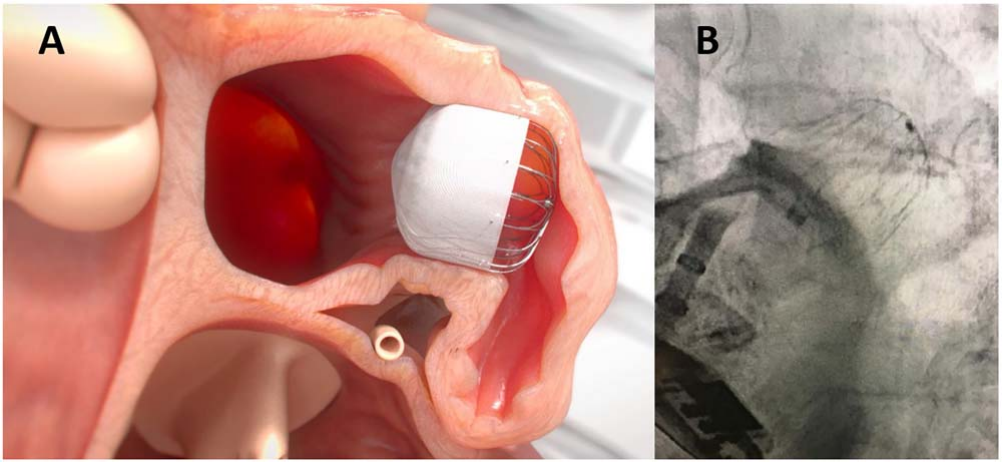
Positioning of a Watchman LAAC device in the left atrial appendage. (A) Simulation of appropriate deployment of a Watchman FLX (Boston Scientific). (B) Fluoroscopic image of a Watchman FLX deployed in the LAA. The deployment catheter has been detached from the device. Abbreviations: LAAC = left atrial appendage closure; LAA = left atrial appendage.

In stroke patients with a history of serious bleeding, who are non-compliant to OAC, or in whom OAC treatment failed to prevent a stroke, an interventional, non-pharmacological technique may be considered to improve stroke prevention (Figure 1, Visual abstract). Closure of the left atrial appendage (LAA),^12^ where most thrombi develop in patients with AF, can be achieved using a percutaneous catheter-based procedure known as left atrial appendage closure (LAAC) or LAA occlusion, employing various devices, one of which is illustrated in Figure 2. Percutaneous LAA ligation can also be performed, and surgical LAA excision/ligation can be done in AF patients undergoing a surgical cardiac intervention.

Left atrial appendage closure is being increasingly offered in many countries^13^^,^^14^ as a second-line therapy for stroke prevention or as an option for AF patients unable to take OAC.^15^ Those caring for stroke patients with AF should appreciate the value and applicability of LAAC. This Practical Guide, written by an international multidisciplinary group, summarising the main results of an international consensus paper and aims to provide a practical overview of the principles, patient selection according to current evidence, peri-procedural complications, post-procedure follow-up and limitations of LAAC, focused on the needs of the stroke physician.^16^

## Evidence for catheter-based LAAC

The efficacy and safety of LAAC were first assessed in the randomised PROTECT-AF^17^ (data collection starting in 2005) and PREVAIL^18^ (starting in 2010) trials in which together randomised 1114 AF patients to either LAAC with the Watchman® device (with warfarin and aspirin for at least 45 days after the procedure) or warfarin (target international normalised ratio of 2–3). After a 5-year follow-up, LAAC provided stroke prevention comparable to VKA in a cohort with 23% prior stroke or TIA at randomisation, with a significant reduction in major bleeding, haemorrhagic stroke, disabling/fatal stroke, cardiovascular (CV) death and all-cause death. However, there was a non-significant trend towards a higher rate of ischemic stroke or systemic embolism (SE) >7 days after the procedure (rate ratio 2.2, 95% credible interval 0.8–4.9).^19^ The PRAGUE-17 trial (data collection from 2015–2019) compared LAAC (using the Amulet or Watchman devices) with OAC, predominantly DOAC, in 402 AF patients (32% with prior stroke), demonstrating the non-inferiority of LAAC relative to OACs in the prevention of stroke/TIA (hazard ratio 1.13, 95% CI, 0.44–2.93), as well as CV death and clinically relevant bleeding over 4 years.^20^

A meta-analysis of PROTECT-AF, PREVAIL and PRAGUE-17 showed a numerically, but non-significantly higher risk of ischemic stroke and SE (risk ratio 1.48, 95% CI, 0.89–2.46) associated with LAAC use (5.6%) relative to OAC (3.6%).^18^ The possibly higher ischemic stroke rate may be explained, for example, by thrombus developing on the LAAC device, air embolism,^21^ thrombus in the LA body due to atrial cardiomyopathy, or because of thrombus or plaque outside the LA. A retrospective multicenter analysis suggests that strokes in AF patients with LAAC at the time of stroke were equally severe on hospital admission, but mortality and severe disability were lower at 3 months compared to AF patients receiving a DOAC.^22^ Additionally, AF patients with LAAC more often underwent thrombolysis than those receiving a DOAC.^22^ In a recent network meta-analysis involving randomised studies comparing LAAC versus OACs (either DOACs or VKAs), and DOACs vs VKAs, there were no significant differences between therapies in the primary endpoint of stroke or SE, or major bleeding. After exclusion of post-procedural bleeding, haemorrhagic risk was significantly lower with LAAC therapy than with DOACs (OR 0.55, 95% CI, 0.35–0.88) or VKAs (OR 0.44, 95% CI, 0.28–0.69).

The OPTION trial compared LAAC vs anticoagulation in 1600 patients undergoing catheter ablation for AF.^23^ The primary efficacy endpoint, which tested for noninferiority, was a composite of death from any cause, stroke or SE at 36 months. The primary safety endpoint, assessed for superiority, was non-procedure-related major bleeding or clinically relevant nonmajor bleeding. LAAC resulted in a non-inferior rate of primary efficacy endpoints (5.3 vs 5.8%, *P* for noninferiority <.001) and a lower rate of primary safety events (8.5% vs 18.1%, *P* for superiority <.001).

Multiple observational studies and registries (eg, ACP registry,^24^ Amulet Observational Study,^25^ EWOLUTION,^26^ NCDR-LAAO registry,^14^^,^^27^ PINNACLE FLX^28^) have evaluated various LAAC devices (ACP, Amulet, Watchman, Watchman FLX, LARIAT)^29^ and the changing profile of AF patients undergoing the procedure, with fewer frail and less comorbid patients.^30^ Many other devices are also undergoing clinical evaluation. The results of these studies suggest that LAAC in AF patients is associated with a 60%–80% lower risk of ischemic stroke and major bleeding relative to expected event rates based on the CHA_2_DS_2_-VASc and HAS-BLED score values at baseline. However, several sources of bias (ie, selection, event ascertainment) and the observational nature of these studies limit their impact on decision-making processes.

At least 5 ongoing clinical trials compare LAAC vs antithrombotic therapy (either antiplatelet agents or OACs) or no antithrombotic therapy in AF patients with elevated bleeding risk, including STROKECLOSE, ASAP-TOO, CLOSURE-AF, CLEARANCE and COMPARE LAAO (Table 1). Five additional trials, including OCCLUSION-AF, CATALYST, CHAMPION-AF, LAAOS 4 and ELAPSE (Table 1), compare LAAC (± DOAC) to DOAC alone in patients with a high risk of stroke or SE. STROKECLOSE and CLEARANCE include patients with a previous ICH, while OCCLUSION-AF focuses only on patients with a previous ischemic stroke or TIA. ELAPSE is enrolling AF patients with a so-called breakthrough stroke (ischemic stroke despite anticoagulation). All patients referred for LAAC should be encouraged, when possible, to participate in a prospective study or registry (Figure 1, Visual abstract).

**Table 1:** Ongoing clinical trials of LAAC against the standard of care

	**CLOSURE-AF**	**STROKE-CLOSE**	**CLEARANCE**	**LAA-KIDNEY**	**OCCLUSION-AF**	**CHAMPION-AF**	**CATALYST**	**LAAOS 4**	**ELAPSE**
Patient population	AF and high bleeding risk (HAS-BLED ≥3 or prior major bleeding; high risk of stroke)	AF and ICH within 12 months	AF and ICH or intracerebral amyloid vasculopathy	AF and end-stage kidney disease	AF and previous ischemic stroke	CHA_2_DS_2_-VASc ≥2 (men) CHA_2_DS_2_-VASc ≥3 (women)	CHA_2_DS_2_-VASc ≥3 initially, now updated to CHA_2_DS_2_-VASc ≥2 (men) CHA_2_DS_2_-VASc ≥3 (women)	CHA_2_DS_2_-VASc ≥4 & either (1) persistent or permanent AF OR (2) Paroxysmal AF with a history of ischemic stroke or SE	Stroke despite active OAC in patients with any form of AF
Number of patients	1000	600	530	430	750	3000	2650	4000	482
Randomisation	LAAC vs best medical care	Amulet vs best medical care (2:1)	Watchman FLX vs best medical care	Amulet vs best medical care	LAAC vs DOAC	WM FLX vs DOAC	Amulet vs DOAC	WM FLX + OAC vs OAC	LAAC+DOAC vs DOAC
Primary endpoint	Stroke, SE, major bleeding, or CV death at 2 years	Stroke, SE, major bleeding, or all-cause mortality at 2 years	Stroke, SE, major bleeding, or CV death at 3 years	Time to first stroke, SE, CV death or major bleeding	Combined rate of stroke, SE, major bleeding and all-cause mortality.	Stroke, SE or CV death at 3 years (non-inferiority) Major or clinically relevant bleeding at 3 years (superiority)	Stroke, SE, or CV at 2 years (non-inferiority) Major or clinically relevant bleeding at 2 years (superiority)	Ischemic stroke or SE (superiority)	Composite of recurrent ischemic stroke, SE, or CV death

Abbreviations: APT = antiplatelet therapy; CV = cardiovascular; CHA_2_DS_2_-VASc = congestive heart failure, hypertension, age ≥ 75 years, diabetes mellitus, stroke, vascular disease, age 65–74 years, sex category (female); DOAC = direct oral anticoagulant; ICH = intracerebral haemorrhage; LAAC = left atrial appendage closure; OAC = oral anticoagulants; SE = systemic embolism; WM-FLX = Watchman FLX.

## Evidence for surgical LAAC

About one-third of patients undergoing cardiac surgery have AF,^31^ and most of these patients are indicated for OAC. The 1:1 randomised LAAOS III study compared best medical therapy versus surgical LAAC in addition to OAC amongst AF patients with a CHA2DS2-VASc score of ≥2 undergoing a planned cardiac surgical procedure for another indication. Anticoagulation was continued in 77% of 4811 participants, and 9% had a prior stroke at the time of enrolment. LAAOS III showed a significant 33% reduction (hazard ratio 0.67; 95% CI, 0.53–0.85) in the risk of stroke/TIA after 3 years.^32^ Perioperative bleeding, heart failure or death rates did not differ significantly between groups. A meta-analysis including previous RCTs and observational studies revealed similar findings.^33^ The LAACS-2 is currently enrolling patients with and without AF, planned for open-heart surgery to surgical LAAC or standard of care (eg, anticoagulation, rhythm control).^33^

## Indications for LAAC

**Figure 3:**
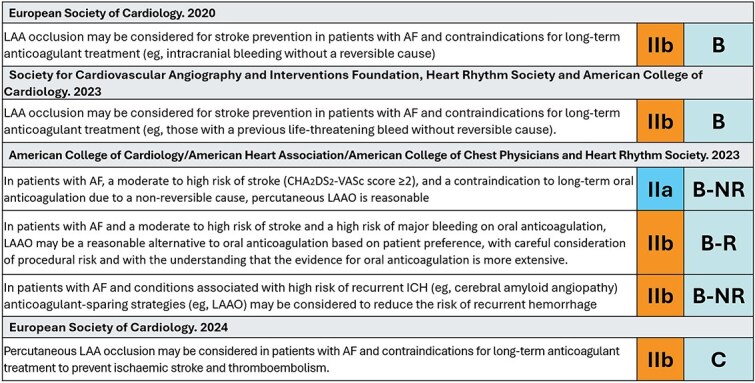
Recommendations for the use of LAA closure in international guideline documents. Abbreviations: LAA = left atrial appendage; ICH = intracerebral hemorrhage; B-NR = level of evidence B according to non-randomised data; B-R = level of evidence B according to randomised data.^3^^,^^6^^,^^34^^,^^35^

Recommendations for the use of LAAC in international guidelines have focused on AF patients with contraindications for (long-term) OAC, even though there are no data supported by RCTs, resulting in a weak class of recommendations, with a low level of evidence (Figure 3).^3^^,^^36–42^ The recent *European Society of Cardiology* AF guidelines state that “percutaneous LAA occlusion may be considered in patients with AF and contraindications for long-term anticoagulant treatment to prevent ischemic stroke and thromboembolism” [class IIb, level C]. However, in American professional societies guidelines, there is an increased class of recommendation for patients with contraindications for OAC (class IIa, level B-NR) and a second recommendation for patients who prefer LAAC as an alternative to OAC (class IIb, class B-NR). However, a recent World Stroke Organization Brain & Heart Task Force scientific statement on the management of AF in patients with a prior stroke or TIA states that “there are no RCTs or subgroup analyses from RCTs in patients with a recent or remote ischemic stroke, TIA or intracranial hemorrhage” supporting the use of LAAC in this population (level of evidence C3).^43^ SCAI and HRS have now issued a recommendation that supports the use of LAAC rather than no therapy in patients with nonvalvular AF with contraindications OAC (conditional recommendation, very low certainty evidence).^44^

Consensus documents have extended consideration for LAAC^34^^,^^45^ in AF patients who: suffer major bleeds or ischemic strokes on optimal anticoagulant treatment, those who refuse or are non-adherent or non-persistent with anticoagulant therapy, those undergoing catheter ablation resulting in the reduction of AF burden to very low levels, or in whom the LAA becomes electrically isolated^35^ and those with clotting disorders incompatible with anticoagulation. As evidence from RCTs is missing besides the OPTION trial,^22^ careful individual risk–benefit assessment and shared decision-making should be undertaken in such patients.^46^ Regarding the recommendation of extending the LAAC indication to AF patients undergoing left atrial catheter ablation, it must be noted that rhythm control does not replace oral anticoagulation, and as such, patients with an at least moderate stroke risk profile still need either oral anticoagulation or LAAC for stroke prevention after ablation.

In the recent European Society of Cardiology AF guidelines,^4^ the results of LAAOS III prompted updated recommendations. Now, “Surgical closure of the left atrial appendage is recommended as an adjunct to oral anticoagulation in patients with AF undergoing cardiac surgery to prevent ischemic stroke and thromboembolism [class I level B] and “may also be considered in patients with AF undergoing endoscopic or hybrid AF ablation to prevent ischemic stroke and thromboembolism” [class IIa level C]. Furthermore, “stand-alone endoscopic surgical closure of the LAA may be considered in patients with AF and contraindications for long-term anticoagulant treatment to prevent ischemic stroke and thromboembolism” [class IIb, level C].

The indication for LAAC should be discussed with the interventional cardiology and/or electrophysiology team. In some healthcare systems (eg, the National Institute for Health and Care Excellence [NICE] in the United Kingdom) insists that a multidisciplinary team selects patients for LAAC^47^ (Figure 1, Visual abstract).

### Procedural issues regarding catheter-based LAAC

The selection of a specific LAAC device and its size depends on the operator’s experience and the LAA anatomy. The pre-procedural diagnostic workup usually includes transesophageal echocardiography or cardiac CT to delineate the LAA anatomy and its suitability for LAAC, and to rule out LAA thrombosis, which can also be done at the beginning of the procedure using intracardiac echocardiography.^48^^,^^49^ Usually, LAA thrombus is considered a contraindication to LAAC, although there are several case series of LAAC when a thrombus is present only in the distal part of the LAA.^50^

If the patient is on a DOAC, the treatment may be stopped a day before the procedure without bridging, but continuous OAC therapy also appears to be safe and effective.^51^ LAAC is often undertaken under general anaesthesia and in some cases with local analgesia and light sedation. Transseptal puncture via femoral venous access is crucial to access the left atrium and deploy an LAAC device. The procedure is guided by echocardiography, and device deployment may be controlled by fluoroscopy.

**Figure 4:**
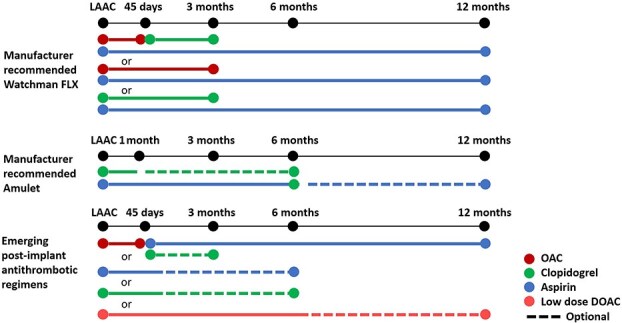
Manufacturer-recommended anti-thrombotic regimens after LAAC and adapted, updated and emerging strategies for anti-thrombotic regimens after LAAC^52^^,^^53^ (limited evidence and some ongoing studies), adapted from.^16^ Abbreviations: LAAC = left atrial appendage closure; OAC = oral anticoagulant; DOAC = direct oral anticoagulant.

Antithrombotic therapy (Figure 4) is required after LAAC to prevent device-related thrombus formation.^46^^,^^54^^,^^55^ The rationale for choosing between the available options should be based on physician assessment of individual patient characteristics, such as bleeding risk and stroke risk, an overall clinical evaluation of the patient’s condition, comorbidities and preference, as well as an evaluation of the reasons for LAAC.^15^^,^^46^^,^^56^ Endocarditis prophylaxis is recommended for 6 months after the procedure.

A network meta-analysis of 41 studies, including 12 451 AF patients undergoing LAAC, suggests that DOACs alone are the antithrombotic therapy associated with the lowest risk of periprocedural thromboembolic events and major bleeding (ranking probability of different antithrombotic regimens estimated based on *P*-scores.^57^ No data from RCTs comparing different antithrombotic strategies are available. The FADE-DRT trial (NCT04502017) is currently comparing aspirin plus clopidogrel, genetically guided antiplatelet therapy and half-dose DOACs for the prevention of stroke, SE and device-related thrombosis in patients undergoing LAAC. Notably, LAAC followed by low-dose aspirin or even no antithrombotic treatment was investigated in the international multicentre *Left Atrial Appendage Occlusion in Patients with Gastrointestinal or IntraCranial Bleeding* (LOGIC) registry Rothwell, 2003 #12829. Furthermore, low-dose aspirin for 3–6 months was an option for LAAC patients at very high risk of bleeding in the randomised CLOSURE-AF trial, which will be presented in a few months.

More details on procedural issues of catheter-based LAAC can be found in an international consensus paper, recently published by the authors.^16^

## Peri- and post-implantation complications

When implantation is performed by experienced implanters using modern devices and adequate imaging at specialised centres, LAAC is a relatively low-risk procedure (Supplementary Table S1).^52^^,^^53^^,^^58–60^ In general, complications occur more commonly in patients with a higher CHA2DS2-VASc score.^61^

*Device embolization* has become a rare complication with the most recent LAAC devices, if device under-sizing, a very proximal implantation, and misalignment of the device to the axis of the LAA are avoided. Percutaneous retrieval is usually successful with a snare catheter or retrieval forceps, but surgical removal from cardiac chambers might be required in some cases.

*Device-related thrombus* occurs in between 2% and 4%, although recent data with newer devices have reported a lower incidence of 1%–2% per year.^62–71^ Device-related thrombosis is associated with a 4–5-fold higher risk of stroke/TIA.^72^ However, as new devices coated with thromboresistant fluorinated polymers are introduced, device-related thrombosis may become less frequent and possibly result in simpler post-implant antithrombotic therapy (eg, DOAC monotherapy in patients with lower bleeding risk or single antiplatelet therapy in patients with high haemorrhagic risk).^73^ Management of device-related thrombosis usually implies low molecular weight heparin or DOACs, but this may be challenging in patients at high bleeding risk. The common practice is to minimise time on anticoagulants until thrombus resolution is verified by advanced cardiac imaging.

As the anatomy of the LAA is highly variable, including the most often non-circular landing zone for the device, there is a risk of *peri-device leak* after implantation.^74^ With current procedural techniques and devices, small peri-device leaks are rather frequent, whereas moderate leaks (3–5 mm) are less common, and severe leaks (>5 mm), which may require interventional leak closure, are rare.^75^ Of note, even moderate peri-device leaks are associated with the composite endpoint of stroke, SE or CV death, as demonstrated in the Amulet IDE trial.^76^^,^^77^

**Figure 5:**
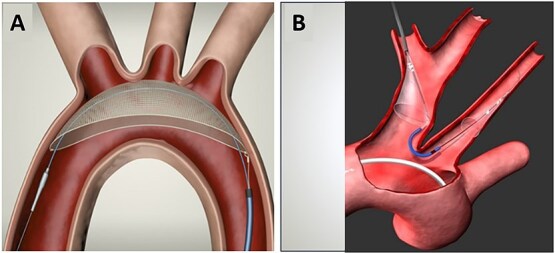
Cerebral protection filter systems. Diagram illustrating positioning of the (A) TriGuard 3 device (keystone heart, Tampa, Florida) and (B) sentinel™ cerebral protection filter system (Boston Scientific, Marlborough, MA, United States).^16^ These devices are designed to protect the cerebral vasculature from embolic events and remove debris/thrombus during interventional procedures, such as Transcatheter Aortic Valve Implantation (TAVI), but have been used for LAAC in patients with thrombus formation in the LAA. Abbreviations: LAAC = left atrial appendage closure; LAA = left atrial appendage.

*Peri-procedural stroke* is mainly due to air embolism or thrombus embolism from the delivery system or implanted device, related to the presence of thrombus/smoke in the left atrium or LAA In randomised trials (PROTECT-AF, PREVAIL and PRAGUE-17), the peri-procedural stroke rate was 0.7%–1.1%, but a peri-procedural stroke rate of 0%–0.2% has been reported in recent multicentre registries (Supplementary Table S1). Cerebral protection systems (Figure 5) are not used as standard clinical practice but may be considered in high-risk cases.^78^ No systematic neurological examination has been performed after the procedure.^79^

There is a risk of clinically unapparent (so-called “silent”) stroke during the procedure, which can be detected by diffusion-weighted imaging. Some studies have reported at least a single acute ischemic brain lesion in 5%–52%.^80^ However, there has been no demonstration of an association of MRI-detected ischemic brain lesions with cognitive decline, as 1 single-centre study reported the Montreal Cognitive Assessment (MoCA) test to be similar in patients with or without MRI-detected ischemic brain lesions after LAAC.

In summary, the safety of implanting an endocardial LAAC device has greatly improved is now as safe as other interventional cardiac procedures.

## LAAC in patients presenting to neurologists

Left atrial appendage closure therapy may offer an advantage over OAC in a variety of circumstances (Figure 1, Visual abstract).

### Intracranial haemorrhage

Stopping OAC and antagonising the anticoagulant effect in patients with acute ICH is a guideline-recommended strategy to reduce ICH-associated morbidity and mortality regardless of the presence of AF and the associated thromboembolic risk. A surgical procedure may occasionally be selected in specific ICH patients.^81^ The residual risk of ischemic stroke in non-anticoagulated AF patients is up to 15% per year, and about 80% of all ICH patients with AF are at high risk of ischemic stroke. This underscores the need to manage thromboembolic stroke prevention after ICH.

Current evidence for restarting OAC after ICH is mainly based on prospective cohort studies and three RCTs, APACHE-AF,^82^ SoSTART,^83^ NASPAF-ICH^84^ and PRESTIGE-AF,^85^ including no more than 660 patients in total.^86^ Taking all RCTs together, re-starting OAC was associated with reduced risk of ischemic stroke on the one hand, but increased risk (of borderline significance) for recurrent intracranial bleeding (ICB).^87^ The threat of ICH recurrence is daunting, but many physicians will consider restarting anti-thrombotic therapy at least 30 days after the ICH.^88^ The results of ongoing RCTs focusing on OAC vs no anticoagulation (without considering LAAC) in ICH patients with AF (such as ENRICH-AF,^89^ PRESTIGE-AF,^90^ A_3_ICH,^91^ STATICH^92^ and ASPIRE^93^) are awaited. In the meantime, there is a specific group of patients who may benefit most from LAAC, given their exceedingly elevated risk of ICH recurrence when OACs are restarted. This specific group includes individuals with large lobar ICH, convexial subarachnoid haemorrhage or cerebral amyloid angiopathy.^94^

Despite the current lack of RCT-proven benefit of LAAC in ICH patients, LAAC is recommended by several AF guidelines^3^^,^^95^ (Figure 3) and consensus papers worldwide,^51^ but the World Stroke Organization assessment adds that more evidence is needed before any strong recommendation can be given.^43^

Publications based on propensity-score matched analyses in AF patients with ICH undergoing LAAC vs medical treatment conclude a benefit of LAAC regarding the composite of ischemic stroke, major bleeding and all-cause mortality.^96^^,^^97^ At present, moderate-sized RCTs comparing LAAC to OAC/best medical treatment, exclusively including ICH patients, such as CLEARANCE,^98^ and STROKECLOSE,^99^ or patients at very high risk of bleeding, including ICH patients, such as CLOSURE-AF^100^ are ongoing (Table 1). Special attention has to be paid to ICH patients with (suspected) cerebral amyloid angiopathy, refractory hypertension or concomitant chronic renal failure (including those on dialysis), who might benefit most from LAAC, and such studies are underway (SAFE LAAC CKD,^101^ LAA-KIDNEY^102^; Table 1).

In clinical practice, LAAC after ICH has “an acceptable peri-procedure and post-procedure risk” according to expert consensus.^103^ Of note, restarting antiplatelet therapy (as needed after LAAC) may be safe after ICH, as demonstrated in the RESTART study, randomising patients on antithrombotic therapy for the prevention of occlusive vascular disease at the time of ICH to restarting or avoiding antiplatelet therapy.^103^ However, it remains to be established in RCTs such as CLOSURE-AF whether stopping antiplatelet(s) several months after LAAC is safe or associated with increased risk of thrombus formation and (subsequent) stroke in AF patients and prior ICH.

To the best of our knowledge, there is no high-quality data regarding LAAC in non-ICH patients with cerebral microbleeds, which can be found in almost 1 out of 4 AF patients and are attributed to cerebral amyloid angiopathy. As the risk of ICH is related to the burden of cerebral microbleeds, further studies are needed to address the issue of LAAC in such patients.^104^

### Ischemic stroke in AF patients while on an OACs

There is scarce evidence regarding the efficacy and safety of LAAC compared to OAC in secondary stroke prevention. RCTs focusing on LAAC vs medical therapy (PROTECT-AF, PREVAIL and PRAGUE-17) and even large prospective LAAC-registries (such as LAARGE, EWOLUTION and AMULET observational registry (Table 1) did not focus on AF patients after ischemic stroke.

The residual stroke risk in anticoagulated AF patients is about 1%–2% per year in RCTs. In the prospective Berlin AF Registry, about 60% of all registry patients with known AF were on OAC at the time of the index stroke or TIA.^105^^,^^106^ Underdosing of DOAC/VKA or a competing stroke aetiology (besides AF) is a frequent finding in AF patients with acute ischemic stroke or TIA.^106^^,^^107^ However, a pooled observational cohort study underlines that about half of all AF patients with ischemic stroke while taking an OAC are neither underdosed nor have a competing stroke mechanism.^107^

As demonstrated by the COMBINE-AF investigators,^108^ and by the multi-centre observational RENO-EXTEND study,^109^ there is a relevant recurrent stroke risk (up to 9% per year) and a rather high mortality rate after ischemic stroke while on OAC. A pooled analysis of observational cohort studies did not demonstrate a benefit of switching the type of OAC,^110^ changing DOAC treatment, or adding an antiplatelet to an OAC^107^ in secondary stroke prevention. Therefore, AF patients suffering an ischemic stroke while on DOAC therapy (properly dosed and taken adherently) are a call to A-C-T-I-O-N,^35^ indicating:

- Aetiology of stroke revisited?
- Compliance to oral anticoagulation optimised?
- Therapeutic options in secondary stroke prevention personalised?
- Intake and interactions for stroke or death treated?
- Novel stroke prevention therapies available.

An important consideration is an alternative or additional thromboembolic protection with novel stroke prevention strategies such as LAAC.

Because of a significant residual risk of stroke under anticoagulation (that may be estimated to be 7% at 1 year and 10% at 2 years), novel stroke prevention strategies may include LAAC on top of oral anticoagulation (and not (dual) antiplatelet treatment), as this combines treatment options in stroke prevention in AF (see Table 1 for details).^108^ An international collaboration of LAAC registries (STR-OAC LAAO) revealed that LAAC in AF patients with a thrombotic event while on OAC led to a similar stroke rate as in AF patients with OAC contraindication undergoing LAAC (EWOLUTION registry).^111^ In addition, in a propensity score-matched comparison between AF patients treated with LAAC versus standard-of-care therapy, LAAC was associated with fewer subsequent ischemic strokes.^112^ LAAC on top of OAC therapy may also be worth considering in light of the results of the LAAOS III trial demonstrating the risk reduction of stroke and SE after surgical LAAC in AF patients undergoing heart surgery and continuing OAC afterwards.^32^ Prospective RCTs using catheter-based LAAC on top of OAC vs OAC are underway (LAAOS-4^113^; and ELAPSE,^114^  Table 1). Further novel prevention strategies may include early rhythm-control therapy in addition to OAC,^115^ left atrial catheter ablation on top of DOAC treatment (as in the ongoing randomised STABLED trial,^116^ bilateral permanent percutaneous carotid artery filter^117^ on top of DOAC treatment (as in the ongoing randomised INTERCEPT trial^118^).

### LAA thrombus despite optimal OAC

Despite optimal OAC treatment, thrombus formation may be detected in the LAA. In a recent meta-analysis, the prevalence of left atrial thrombus in patients with AF or atrial flutter during optimal anticoagulation was 2.7%, regardless of whether patients were treated with a VKA or DOACs.^119^ Current recommendations suggest that LAAC should not be performed in such cases because of the risk of provoking dislodgement of the thrombus and subsequent embolism. Therefore, LAAC use in such cases is anecdotal.^120^^,^^121^

### Severely reduced glomerular filtration rate and kidney failure

The prevalence of AF is high in patients with an estimated glomerular filtration rate (eGFR) <30 mL/min or undergoing dialysis. RCTs demonstrating the efficacy of per se contraindicated VKA for thromboembolic prevention in AF are lacking, and observational studies in haemodialysis patients have yielded uncertain results.^122^ Two recent meta-analyses of studies performed in severely reduced glomerular filtration and kidney failure populations were unable to demonstrate that OAC therapy was associated with a reduced risk of thromboembolism.^123^^,^^124^ Two RCTs evaluating the safety of LAAC vs OAC therapy in patients with eGFR <30 mL/min WATCH AFIB^125^ and STOP-HARM,^126^ were terminated prematurely due to failure to recruit patients.^127^ Other RCTs, such as LAA-KIDNEY^102^ and SAFE-LAAC CKD (NCT05660811) are ongoing.^16^

### Patient refusal/non-adherence/non-compliance

Left atrial appendage closure may be a valuable alternative for treating patients refusing OAC or who frequently lapse from their therapy.^126^^,^^128–130^ The 2023 ACC/AHA/ACCP/HRS guidelines do accept that patient preferences may be considered [class IIb recommendation].^42^ Moreover, physicians may decide not to prescribe DOAC in elderly and/or frail patients or in patients who need these medications, but have important drug–drug interactions. Notably, there are some reports on LAAO in octogenarians, reporting a similar safety profile and comparable efficacy to non-octogenarians.^16^ If LAAO becomes a standard of care for stroke prevention, it will be essential to consider the expected lifespan of patients being evaluated for the procedure. Current clinical trials will help determine the minimum survival duration needed to derive benefit from LAAO, similar to previous findings from carotid endarterectomy trials,^130^ where the efficacy of the intervention was contingent upon projected survival.

## Conclusion

For primary stroke prevention, LAAC is nearly as effective and is safer than VKA therapy. However, data derived from RCTs comparing DOACs and LAAC are still insufficient to justify considering LAAC as first-line therapy. Several RCTs are ongoing, and the indications for LAAC are likely to be extended shortly, particularly to include high-risk groups for stroke, such as patients with anticoagulant-related ICH, ischemic stroke despite anticoagulation and patients who cannot or will not take OACs. RCTs assessing the value of LAAC versus OAC, or as a hybrid approach on top of OAC, are ongoing, and the results are eagerly awaited, as they will fill gaps in evidence and may satisfy unmet needs.

Neurologists and stroke physicians may consider LAAC in selected patients, but should be aware of potential complications and present limitations. Stroke patients more likely to benefit are those with a previous large lobar ICH, convexial subarachnoid haemorrhage or cerebral amyloid angiopathy due to the reported exceedingly elevated risk of recurrent ICH. Patients with multiple stroke recurrences, despite good compliance with anticoagulation and no evidence of competing mechanisms, may be potential candidates for LAAC. At this stage, enrolment in clinical trials is also highly recommended to enrich the evidence base.

## Supplementary Material

aakaf018_ID_ESO-25-0511_R4_SUPPLEMENTAL_MATERIAL_highlighted_changes

## Data Availability

Not applicable.
