# Supplementary material for: Update on left atrial appendage closure for neurologists
Source: Eur Stroke J. 2026 Jan 1;11(1):aakaf018. doi: 10.1093/esj/aakaf018 (PMC12866672; doi:10.1093/esj/aakaf018)
Supplement: aakaf018_ID_ESO-25-0511_R4_SUPPLEMENTAL_MATERIAL_highlighted_changes [file aakaf018_id_eso-25-0511_r4_supplemental_material_highlighted_changes.docx]

**SUPPLEMENTAL MATERIAL**

**Supplemental Table S1: Incidence of peri-procedural LAAC complications in approval studies and real-world experience**  *^60,68,76,X1,X2^*

|  | **WATCHMAN FLX** | | **AMPLATZER AMULET** | |
| --- | --- | --- | --- | --- |
|  | **Pinnacle FLX** **(approval study)** ^68^  **N = 400** | **SURPASS**  **(RWE)**  ^X1^  **N = 97,185** | **Amulet IDE (approval study)**  ^60, 76^  **N = 915** | **EMERGE**  **(RWE)**  ^X2^  **N = 5,499** |
| Procedural Success | 99% | 98% | 98% | 96% |
| Safety Composite Endpoint* | 0.5% | 0.45% | 1.5% | 0.8% |
| **Procedural Safety Events (7dd or hospital discharge, whichever is later)** | | | | |
| Pericardial tamponade/effusion | 0.0% | 0.39% | 2.4% | 1.3% |
| Device embolization | 0.0% | 0.03% | 0.7% | 0.2% |
| All Stroke | 0.5% | 0.08% | 0.0% | 0.2% |
| Death | 0.0% | 0.09% | 0.0% | 0.40% |
| **45-day Safety Events** | | | | |
| Pericardial tamponade/effusion | 0.7% | 0.44% | NR | 1.9% |
| Device embolization | 0.0% | 0.04% | NR | 0.2% |
| All Stroke | 0.7% | 0.29% | NR | 0.3% |
| Death | 0.5% | 0.81% | ? | 1.0% |
| Device-related thrombus | 0.25% @45dd  1.8% @1yr & 2yr | 0.44% @45dd | 2.4% @45dd  3.3% @ 18mo | NR |
| Complete LAA Closure | 83% @45 dd  90% @1yr | 83% @45dd | 63% @45 dd  63% @1 yr | 87% @45 dd |

*NR = not reported*

**Safety Composite Endpoint: Defined as the occurrence of all-cause death, ischemic stroke, systemic embolism, or device or procedure-related events requiring open cardiac surgery or major endovascular intervention between device implantation and seven days or hospital discharge (whichever is later).*

*Results from different clinical investigations are not directly comparable. Information provided side by side for illustrative purposes only.*

*The NCDR LAAO Registry relies on site-reported data, which may lead to over- or under-reporting of patient, hospital, or physician data. However, the NCDR program includes annual audits of site data collection and a validated computer-based algorithm to adjudicate clinical endpoints to ensure data quality. ^60,68,76,X1,X2^*

X1 - Kapadia SR, Yeh RW, Price MJ, et al. Outcomes With the WATCHMAN FLX in Everyday Clinical Practice From the NCDR Left Atrial Appendage Occlusion Registry. *Circ Cardiovasc Interv* 2024; 17: e013750. 20240726. DOI: 10.1161/CIRCINTERVENTIONS.123.013750.

X2 - Alkhouli M, Freeman JV, Ellis CR, et al. First Experience With Amulet in the United States: Early Insights From EMERGE LAA Postapproval Study. *JACC Cardiovasc Interv* 2024 20240119. DOI: 10.1016/j.jcin.2023.11.027.
